# Nanovesicular Drug Delivery Systems for Overcoming Antibiotic Resistance

**DOI:** 10.7759/cureus.98926

**Published:** 2025-12-10

**Authors:** Gopalakrishna Pillai, Satish Jankie

**Affiliations:** 1 Department of Pharmaceutical Sciences, South University School of Pharmacy, Savannah, USA; 2 School of Pharmacy, The University of the West Indies, Mt. Hope, TTO

**Keywords:** drug delivery, liposomes, nanodrug delivery systems, nanovesicles, niosomes, surfactant nanovesicular systems, targeted drug delivery systems

## Abstract

Despite the early success of treating bacterial infections with antibiotics, resistance development has become a global problem. Treatment of organisms that have become resistant to common antibacterial agents is challenging and can cause severe and extended illness, longer hospital stays, and increased medical costs. Bacteria may develop antibiotic resistance by multiple biochemical pathways, including target-site gene mutations, decreased permeability, drug uptake or efflux mechanisms that remove the drug from the host cells, and alterations in metabolic pathways. Several strategies to overcome antibacterial resistance are under investigation, such as modifying existing antibiotics, targeting bacterial enzymes, cell-penetrating antibiotics, nanoparticle drug delivery systems, and encapsulating antibiotics and macrophage-targeted nanoparticles conjugated with specific ligands. Among all these approaches, the nano-sized drug delivery systems have become the subject of intense investigations because of their special benefits, including increased stability of the antibiotic, enhanced permeation through cell membranes, and delivery inside the cytoplasm, as well as improved biodistribution and pharmacokinetics. This review compiles data on liposomes and niosomes, promising nanovesicular drug delivery systems capable of targeting antibiotics to fight resistant microorganisms.

## Introduction and background

Although antibiotics have changed the landscape of medicine by reducing mortality, their effectiveness is being challenged by the rapidly increasing numbers of multidrug-resistant pathogens. Life-threatening infections due to resistant organisms and the associated financial burden to the healthcare budget are pressing concerns [1]. Antibiotic resistance is problematic as it decreases viable therapeutic options and delays efficacious treatment, resulting in severe and extended illness, longer hospital stays, reduced quality of life, and increased medical costs. Organisms resistant to multiple antimicrobials are rampant in hospital environments and within community settings. Particularly problematic are the major human pathogens, such as *Pseudomonas aeruginosa*,* Klebsiella pneumoniae*,* Acinetobacter baumannii*,* Enterococcus faecium*,* Staphylococcus aureus*,and* Enterobacter spp*. The challenge of treating these organisms has been acknowledged by the World Health Organization (WHO), which has recognized it as a major global and public health threat [2].

**Figure 1:**
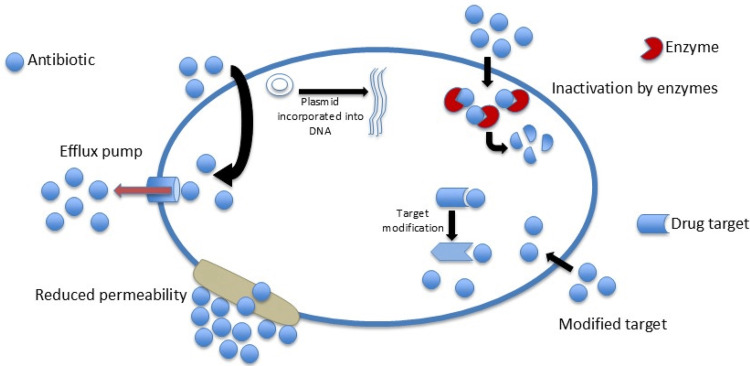
Mechanisms of antimicrobial resistance in the bacterial cell.

Increasing trends in antimicrobial resistance are evident based on the WHO Global Antimicrobial Resistance and Use Surveillance System Report 2022. From 2017 to 2020, data from 76 countries showed an average rate of resistance for *E. coli* resistant to third-generation cephalosporin (42%) and methicillin-resistant *S. aureus* (35%). *E. coli*-induced urinary tract infections showed a 20% reduced susceptibility to ampicillin, co-trimoxazole, and fluoroquinolones [3]. Bacteria have evolved and, through various mechanisms, can withstand threats and thrive in the presence of antibiotics designed to eradicate them. Resistance can develop through multiple biochemical pathways that modify the antimicrobial target, decrease drug uptake, efflux mechanisms that remove the drug from the host cell, and alterations in metabolic pathways [4], as shown in Figure 1.

Fluoroquinolones target the nucleus and must penetrate the outer layers of the bacterial cell to exert their bactericidal properties. The effectiveness depends on the capacity to penetrate cellular barriers and their ability to evade efflux pumps located in the cell walls [5]. Permeability through the outer membrane is important because many agents currently being used have intracellular targets and must breach the bacterial cell membranes to exert their therapeutic effect. Bacteria are now capable of reducing the uptake of antibiotics, thereby preventing them from reaching the intracellular target.

Fluoroquinolone resistance occurs through target-site gene mutations of DNA gyrase and topoisomerase IV in Gram-negative and Gram-positive organisms, respectively. This alters the drug’s ability to effectively bind to its target enzymes. Mutations within the nucleus can lead to reduced uptake by lowering their outer membrane permeability or by increased efflux activity, thereby reducing plasma concentrations within the cell and the effectiveness of the drug [6]. The existence of an efflux pump was first observed in* E. coli* that pumped out tetracycline from its cytoplasm [7]. Currently, various types of efflux pumps have been isolated from Gram-positive and Gram-negative organisms [8]. The AcrAB-TolC pump in *Salmonella* and *E. coli* can confer resistance to fluoroquinolones as well as to other antibiotics [9]. *E. coli*,* S. pneumoniae, *and* S. aureus* are organisms where efflux mutant pumps are frequently recovered in clinical isolates. Bacterial drug efflux inhibitors can overturn multidrug resistance and render antibiotics reusable. Multiple efflux pumps may exist in a single bacterium (e.g., 10 in *Salmonella enterica *and20* in E. coli*) that facilitate the development of natural and acquired resistance in bacteria [10]. Therefore, developing an inhibitor of a single pump may not be a solution to multidrug resistance, and, to date, no clinically useful efflux pump inhibitor has been found.

In Gram-negative organisms, the production of beta-lactamases has been hailed as the major contributory factor for resistance to beta-lactam antibiotics [11]. The mutated beta-lactamases (extended-spectrum beta-lactamases (ESBLs)) have activity against the newly developed beta-lactam antibiotics. Common ESBL-producing organisms include *E. coli* and *K. pneumoniae*, and carbapenemase is produced by carbapenem-resistant *Enterobacteriaceae*. Resistance among Gram-positive bacteria is mostly due to modification of the penicillin-binding protein (PBP) at the target site. Bacteria can develop resistance by reducing access to PBPs, reducing affinity to PBPs, or altering the structure of PBPs. For example, Pneumococci develop resistance to penicillin by altering the structure of PBP. In the case of aminoglycosides (AGs), the most common mechanism is enzymatic deactivation by AG acetyltransferases, AG nucleotidyltransferases, and AG phosphotransferases. AG resistance also occurs by modification of the ribosome target by a family of ribosomal methyltransferase enzymes. Intracellular concentrations of AGs may remain low even after penetration through the cell membrane due to the active expulsion of AGs by the efflux pumps [12].

Biofilms

Biofilms comprise a self-produced extracellular polymeric matrix into which various microorganisms embed themselves. This environment allows the organism to survive and proliferate, tolerating antibiotics and facilitating the development of resistance. The increased selection pressure and the prevailing conditions encourage its growth and determine the composition of the biofilm, its structural design, and stage of development [13]. Biofilm production decreases the antibiotic penetration capacity, reduces intracellular concentrations, and favors the organism developing tolerance to the antibiotic and being able to persist in that environment. In addition, efflux pumps may be expressed in the biofilm, further reducing the likelihood that the drug may reach its target site. A thorough investigation of the processes that allow bacteria to survive in the presence of antibiotics can guide scientists to develop mechanisms to successfully treat these infections [13]. It is reported that antimicrobial agents are 10-1,000-fold less likely to effectively treat an organism in its biofilm form when compared to its planktonic forms [13-15]. Numerous mechanisms are associated with the development of resistance to antibiotics when organisms are in the biofilm configuration. These mutations increase the amount of multidrug-resistant organisms in the biofilm, which acts as a developing pool of antibiotic-resistant genes [16].

Drugs trapped in nanovesicles are superior to the conventional free drug as they reduce the volume of distribution, target the site of drug action, and shield the drug from the external environment. They can circumvent the adverse drug effect profile of a drug by protecting it from the external environment, thereby reducing the chance of an allergic reaction. For instance, in patients treated with vancomycin, there is a risk of a severe hypersensitivity reaction, specifically drug reaction with eosinophilia and systemic symptoms syndrome. This exaggerated immune response can be mitigated by nanovesicles that shield the drug, protecting it from degradation and avoiding interaction with the host immune system [17]. This review examines liposomes and niosomes as promising nanovesicular drug delivery systems to combat antibiotic resistance by enhancing drug stability, membrane permeability, and intracellular targeting, thereby providing critical insights into innovative strategies for revitalizing existing antibiotics against resistant pathogens and addressing one of the most pressing global health challenges of our time.

## Review

Methodology

This review was conducted following an extensive and comprehensive examination of the available literature on nanovesicular drug delivery systems for overcoming antibiotic resistance, with particular emphasis on liposomes and niosomes. We did not establish specific inclusion or exclusion criteria for the literature selection, nor did we impose temporal restrictions on the literature search or perform formal data synthesis, consistent with the narrative review methodology.

The scope of this narrative review encompassed the following key areas: first, we examined the clinical significance of antibiotic resistance as a growing healthcare challenge and public health threat. Second, we evaluated current therapeutic approaches and limitations in addressing multidrug-resistant bacterial infections. Third, we conducted a comprehensive narrative review of recent evidence from preclinical and clinical studies investigating the efficacy of nanovesicular drug delivery systems, particularly liposomes and niosomes, in enhancing antibiotic penetration and overcoming resistance mechanisms. Additionally, this review highlighted the specific advantages of nanovesicular formulations over conventional antibiotic delivery methods, including improved bioavailability, targeted delivery, reduced systemic toxicity, and enhanced therapeutic efficacy against resistant pathogens.

We systematically organized these topics and present our analysis on optimal nanovesicular drug delivery strategies for combating antibiotic resistance, offering insights that may inform future therapeutic developments and clinical applications in this critical area of antimicrobial therapy.

Strategies to overcome bacterial resistance

The use of nanopraticles to treat bacterial infections has shown promise, and its evolution has led to the development of solid lipid nanoparticles (SLNs) and nanostructured lipid carriers (NLCs). SLNs are lipid-based colloidal systems stabilized in an aqueous phase, where the lipid component remains solid under physiological conditions. Both systems offer advantages, including improved solubility, enhanced permeability, greater bioavailability, and prolonged systemic retention, while supporting targeted delivery with minimal toxicity. NLCs differ from SLNs in that they incorporate both solid and liquid lipids within their matrix. This structural modification enables superior drug-loading capacity, controlled release behavior, and improved formulation stability [18]. Compared to organic nanoparticles, inorganic materials exhibit superior stability and diverse physicochemical, mechanical, magnetic, and optical properties. Their surfaces can be functionalized with ligands to improve affinity for specific targets, making them an attractive option for imaging and drug delivery. A key advantage of drug-inorganic nanoparticle conjugates is localized delivery to diseased cells, which has the potential to reduce systemic toxicity and improve therapeutic efficacy [19]. Microfluidics is an important aspect of nanoparticle evaluation as it serves as a platform to replicate complex physiological conditions such as dynamic flow, chemical gradients, and multiorgan interfaces. It offers a cost-effective and precise method for nanoparticle synthesis and evaluation. These systems enable controlled fabrication of nanoparticles, improving consistency across batches [20]. The major methods employed by nanovesicles to combat antimicrobial resistance are discussed further by using suitable examples of liposomes and niosomes.

Targeting the Infection Site

**Figure 2:**
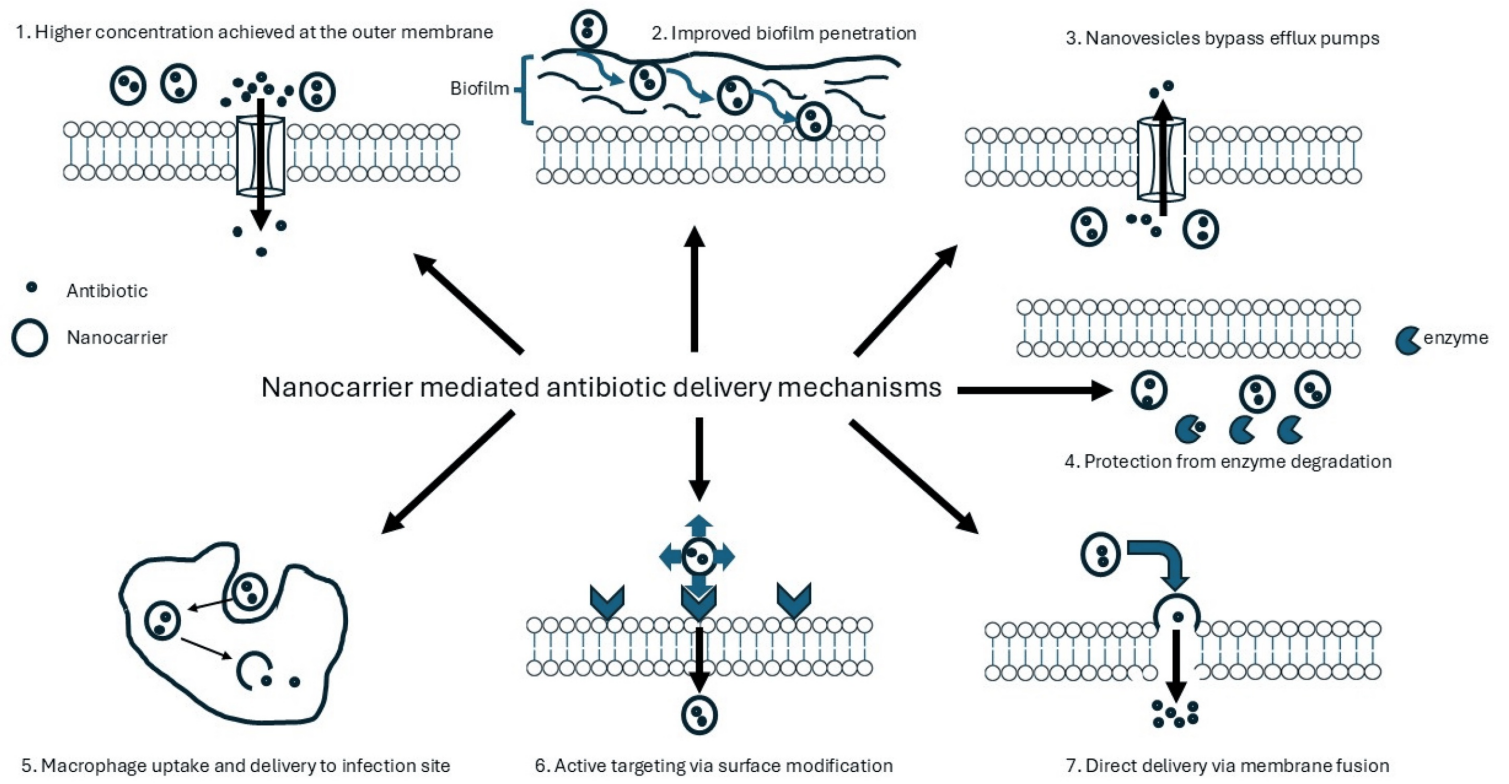
Major mechanisms utilized by nanovesicles to circumvent antimicrobial resistance.

Various methods have been employed to combat the evolution and persistence of multidrug-resistant pathogens (Figure 2). Some of these include dual antibiotic therapy, mechanisms to target the proteins or enzymes liable for the development of resistance, the utilization of modified drug delivery systems, and methods that alter physicochemical properties of the active agent [21]. A strategy to counter resistance is to develop novel drug carriers that would protect the antibiotic from enzymatic attack and deliver the antibiotic inside the cytoplasm without being pumped out by the bacterial efflux pumps. Conventional drug delivery systems have drawbacks, including poor in vivo stability, limited aqueous solubility affecting absorption and bioavailability, and poor membrane permeability to provide effective concentrations intracellularly.

Liposomes

Applying nanotechnology to novel drug delivery systems prevents degradation of the drug and delivers greater quantities of the active agent to the site of action [21]. Nanoparticle drug delivery systems have shown enhanced absorption and bioavailability. This improves the pharmacokinetics and biodistribution, increasing retention in the body and prolonging the drug effect while actively delivering the drug to the desired site. Liposomes (phospholipid vesicles) [22] and niosomes (non-ionic surfactant vesicles) [23] are nanosized spherical vesicles (10-200 nm diameter) composed of an outer bilayer membrane formed with phospholipids/surfactants and cholesterol and an inner aqueous compartment. Hydrophobic materials are trapped in the outer lipid membrane, with hydrophilic materials being enclosed in the inner aqueous compartment.

Liposomes, the forerunners of vesicular systems, have been used successfully to entrap various drug molecules and transport them to the target sites. In 2018, a liposome-antibiotic, Arikayce, was approved by the Food and Drug Administration (FDA) to treat* Mycobacterium avium* complex lung disease [24]. Due to the similarities in the structure and composition between the bacterial cell membrane and the liposome, the liposome may fuse with the cell membrane via endocytosis and deliver a higher concentration of the drug within the bacterial cell. Liposomes have an inherent advantage as they are taken up by macrophages and migrate to the infection site, where they are released. The development of rifampicin [25] and rifabutin-liposome (RFB) [26] for the treatment of *Mycobacterium tuberculosis* intracellular infections is based on this concept. A recent study of liposomal rifabutin has shown the effectiveness of this formulation against methicillin-resistant *S. aureus* (MRSA) as well, especially the more resistant biofilm-organized bacteria. Liposomal RFB was superior to free vancomycin and unencapsulated RFB, with the liposomal formulation exhibiting reduced bacterial counts in major organs [27].

Encapsulation of antibiotics in liposomes offers protection to the drug molecules and prevents them from being degraded, thereby increasing the concentration of the drug at the infection site, a mechanism useful for treating organisms resistant to multiple antibiotics such as MRSA [28]. Commonly used antibiotics that have been encapsulated in liposomes, their objectives, and major findings are shown in Table 1.

**Table 1:** Commonly used antibiotics that have been encapsulated in liposomes for in vitro/in vivo evaluation. MIC = minimum inhibitory concentration; MRSA = methicillin-resistant *Staphylococcus aureus*

Antibiotic	Objective	Major findings	Reference
Polymyxin B	To investigate the antibacterial effects of liposomes loaded with polymyxin B modified with chitosan in vitro	The combination of antibacterial synergetic effect eliminated the biofilm-producing bacterium	[29]
Fusidic acid (FUS)	To investigate the effect of unilamellar vesicles loaded with FUS against different bacterial strains in vitro	Encapsulating FUS in a liposome improved antimicrobial efficacy by reducing the MIC. It also showed efficacy against Gram-negative organisms, while unencapsulated FUS did not	[30]
Vancomycin	To determine if vancomycin encapsulation in liposomes can extend its spectrum of activity to Gram-negative organisms in vitro	The liposomal formulation showed activity against isolates of *Escherichia coli* and *Acinetobacter baumannii*, whereas the free drug did not	[31]
Amikacin, gentamicin, tobramycin	To determine the bactericidal efficacy of liposome-encapsulated formulations against strains of *Pseudomonas aeruginosa*	MICs were maintained or reduced for the liposomal formulation against all tested clinical isolates	[32]
Gentamicin	To determine the effect of gentamicin entrapped in liposomes against *Pseudomonas aeruginosa*	Gentamicin entrapped in liposomes showed lower MIC values compared to free gentamicin. Liposomal gentamicin produced killing times that were less than or equivalent to the free antibiotic	[33]
Norfloxacin	To determine the antimicrobial efficacy of a hybrid liposomal formulation against various organisms, including *Salmonella heidelberg*, *Salmonella typhimurium*, *Campylobacter jejuni*, *Pseudomonas aeruginosa*, and *Escherichia coli*	Liposomal formulations showed a greater antibacterial effect when compared to the free drug	[34]
Ofloxacin	To determine the effect of liposomal ofloxacin activity against and accumulation in bacteria in comparison to that of free drugs	Liposomal formulations showed a 2–4-fold reduction in MICs when compared to the free antibiotic	[35]
Cefepime	To determine the antimicrobial activity of a liposomal formulation of cefepime against *Escherichia coli*	Both formulations showed similar antibacterial activity against the *Escherichia coli* strain	[36]
Azithromycin	To assess the effect of liposomal azithromycin in treating cervicovaginal infections	Liposomal formulations were similar to free antibiotics, but the effect against biofilm-producing organisms was dependent on the lipid composition	[37]
Ciprofloxacin	To determine how encapsulation of ciprofloxacin in liposomes can alter the pharmacokinetic properties of the drug in an in vivo model	The liposomal formulation presented a higher circulation lifetime, and the livers and spleens of infected mice showed 10³–10⁴-fold lower levels than the unencapsulated drug	[38]
Piperacillin	To determine if encapsulation of piperacillin by liposomes protected the drug from hydrolysis by staphylococcal beta-lactamase	Liposomal piperacillin was three times more effective in inhibiting *Staphylococcus aureus* growth than the free drug. It also offered better protection against staphylococcal beta-lactamase hydrolysis	[39]
Vancomycin	To determine if liposomal formulations of vancomycin have superior activity against MRSA when compared to the free drug	Liposomal vancomycin had MICs that were up to 4-fold lower than the free drug. The formulation restricted the growth of MRSA inside macrophages	[40]
Cinnamaldehyde (CA)	To determine the effect of CA-loaded P-Lipo against *Escherichia coli* and *Pseudomonas aeruginosa* and its impact on infected mice	The formulation showed increased delivery of CA into the bacterial cell and improved its antibacterial effect. It reduced the inflammatory response by decreasing proinflammatory cytokine release	[41]

Gram-Positive Organisms

*S. aureus* is the primary source of bacterial infections in tertiary-level hospital settings and in the community. MRSA is a significant challenge in clinical settings, reflected in its persistently high morbidity and mortality rates. They persist even after being engulfed by macrophages, which combat bacterial infections as they recognize, engulf, and break down the invading pathogens [42]. Bacteria engulfed by macrophages act as reservoirs for bacterial reproduction, resulting in recurrent infections and resistance. Bacterial cells that survive within host cells can shield themselves from the antibacterial effects of antibiotics as they are unable to penetrate and be retained within the cells (such as macrolides or fluoroquinolones) [43,44] or because of poor accumulation inside the cells (such as β-lactams or AGs) [45,46]. Although vancomycin is reserved as the final option in treating MRSA, it is unable to eliminate MRSA inside host cells as its accumulation within the cells is limited [47]. Macrophages can be used to deliver antibiotics specifically to infected cells and thereby enhance antimicrobial effects against these infections. *S. aureus* residing intracellularly can exist in a state of dormancy, a protective mechanism, as most antibiotics exert their effect on metabolically active organisms. These organisms cannot be eliminated even with increased uptake and increased intracellular concentrations. Rather, a new treatment option is required that can render activity against “hidden” intracellular organisms.

Lysostaphin

Lysostaphin is an antimicrobial enzyme that specifically targets Gram-positive pathogens in vivo and in vitro [48]. Its activity extends beyond planktonic forms of the organism to dormant organisms lying within a biofilm [49]. It achieves this action by cleaving the pentaglycine cross-links in the cell walls [50], allowing its bactericidal action to proceed regardless of the organism’s metabolic state. Lysostaphin’s activity can be enhanced by utilizing a drug carrier to strategically deliver the drug within the dormant bacterial cell. When lysostaphin is combined with vancomycin, a synergistic effect is observed against extracellular *S. aureus* and MRSA [51]. Therefore, a combination of lysostaphin and vancomycin can enhance the eradication of intracellular bacteria, allow for a reduced dose of vancomycin, and decrease the incidence of toxicity and resistance.

Intravenous antibiotic therapy is used for pulmonary exacerbations in cystic fibrosis and chronic obstructive pulmonary disease patients, but the development of resistance is common. Effective antimicrobial therapy for respiratory tract infections that allows accurate targeting of pathogens such as *P. aeruginosa *and* S. aureus* is urgently required. The pulmonary route may be chosen to treat respiratory infections with the advantage of ease of administration and patient compliance.

Inhalation Therapy

Liposomal antibiotics formulated for inhalation therapy are taken up by the alveolar macrophages with subsequent release of the antibiotics, allowing penetration through the biofilm and increased activity. Aerosol therapy is especially useful for direct delivery of antibiotics that penetrate lung tissue poorly, such as beta-lactams, AGs, glycopeptides, and colistin. Several aerosol antibiotics have been approved because of their reduced systemic toxicity and immunomodulatory effects [27,52-58]. Administration of antibiotics without encapsulation in liposomes or niosomes may result in a short circulation time and rapid removal of the drug from the lungs. To overcome this problem, nanovesicular delivery systems are used to achieve a sustained release of the antibiotic and allow for antibiotic concentrations to be maintained above the minimum inhibitory concentration in a sustained manner with a less frequent dosing interval. Aerosol formulations of antibiotics entrapped in liposomes, such as amikacin, ciprofloxacin, and tobramycin, are being developed to treat infections caused by mycobacteria and *P. aeruginosa* [54]. This strategy improves the efficacy of therapy in managing lung infections caused by multidrug-resistant bacteria, with the option of a single liposome containing two antibiotics also being considered. A dry powder liposomal formulation for inhalation containing ciprofloxacin and colistin has been developed, which demonstrated synergistic effects against *P. aeruginosa* [57].

Topical Delivery

Liposomal antibiotics can also be formulated into ophthalmic and dermal dosage forms. Locally administered topical dosage forms containing antimicrobials are preferred in the treatment of infections caused by burns and chronic wounds secondary to diabetic, vascular, and pressure ulcers [59]. Topical dosage forms face many challenges, such as drug instability and barriers to penetration through the body’s natural defence mechanisms. Multidrug-resistant organisms such as MRSA can penetrate deeper into skin and soft tissues, leading to cellulitis, abscesses, or necrotizing fasciitis. This may require the development of dosage forms that will allow for enhanced delivery and absorption at the site of infection.

Liposomal formulations have been developed for topical skin infections that allow for skin regeneration, wound healing, and pathogen eradication [60]. One such formulation includes the liposomal encapsulation of silver sulfadiazine, which reduced the bacterial counts of *P. aeruginosa* after a single application, compared to the conventional free drug formulation, when tested in soft tissue infection [61]. Liposomal polyvinylpyrrolidone-iodine is a commercially available formulation for the treatment of wounds infected with resistant bacteria. Liposomal polyvinylpyrrolidone-iodine formulation has demonstrated therapeutic benefits for several inflammatory skin disorders [62]. An approach used for targeting antibiotics at the infection site is based on the liposome’s surface charge. A positive charge on the liposome surface can target the negatively charged cell wall of the microorganisms by electrostatic interaction [63-65].

Stimuli-Responsive Liposomes

Liposomes can be developed that allow for drug release when exposed to specific environmental factors such as pH or temperature. pH-sensitive liposomes release their contents when exposed to an acidic pH, achieved through changes in their conformation and chemical structure. One such dosage form utilizes cholesteryl hemisuccinate combined with dioleoylphosphatidylethanolamine in the liposome bilayer, which becomes destabilized in response to an acidic environment, promoting the release of the incorporated molecule. This is particularly useful in treating infectious biofilms, as they are known to possess an acidic pH [60]. Liposomes that are sensitive to changes in temperature release their content when exposed to local heating, which can occur due to local release of histamine [66]. These entrapped drugs are released as the temperature exceeds the melting transition temperature of the bilayer [67].

Targeting Ligands

Liposomes can be prepared by grafting a ligand onto their surface, which would allow for targeting a specific bacterial cell. Some targeting ligands include the use of aptamers, proteins, antibodies, or antibody fragments and polysaccharides. These would be identified by surface receptors located on the target cells. This would achieve transport of the antibiotic to the desired location from the liposomal formulation [68]. Enhanced drug delivery can reduce the total dose administered, which decreases the likelihood of adverse effects and dose-dependent toxicity. Use of ligands in the preparation of liposomal vancomycin reduces the risk of nephrotoxicity, which often limits its clinical usefulness [40]. Similar approaches are used in developing liposomal formulations to treat *M. tuberculosis *or *Listeria monocytogenes* intracellular infections [45,51]. Vancomycin-encapsulated liposome for topical administration showed enhanced drug delivery. Lysostaphin, an endopeptidase enzyme (anti-staphylococcal protein), disrupts the organism by binding to the *S. aureus* cell wall (peptidoglycan moiety). The enzyme was grafted onto the surface of the liposome to target the microbe by conjugation of the cyanuric functional group. The lysostaphin-conjugated liposomes exhibited a higher binding rate (as determined by flow cytometry of the fluorescently labelled liposomes) and antibacterial effect than non-conjugated liposomes. A formulation of lysostaphin and vancomycin to treat MRSA infections utilized a mannose-modified exosome as the drug carrier, allowing for preferential uptake by macrophages. After intravenous administration of the conjugate, vancomycin accumulated in higher concentrations in the spleen and liver, making it useful for targeting intracellular pathogens [69].

Nanoplatforms for Bone Infections

Bone infections are challenging to treat as they require antibiotic use for lengthy periods. A possible cause of therapeutic failure is the reduction in vascular supply to the area subject to necrosis and infected bone, which reduces the ability of antibiotics to achieve inhibitory concentrations at the infection site [70]. Some drug delivery systems associated with medical device implantation for the treatment of bone infections include FDA-approved bone cement and PMMA beads containing gentamicin [71-73], although there is a need to improve antibiotic release from the drug delivery system [74]. MRSA bone infections have been treated with liposomal formulations of gentamicin, vancomycin, ceftazidime, and dicloxacillin [63]. The ability of calcium-based salts to bind to bone resulted in the preparation of liposomes coated with calcium phosphate, allowing for targeted delivery to a specific site [75]. A formulation built on the same concept utilized a calcium phosphate scaffold, which incorporated gentamicin into positively charged liposomes. This resulted in sustained release preparation with superior activity (complete sterilization of bone tissue) when compared to the unencapsulated conventional formulation [76].

Nanoplatforms for Central Nervous System Infections

Therapeutic failure in treating bacterial meningitis can occur if the antibiotic is unable to penetrate the blood-brain barrier (BBB). One strategy to circumvent this is to administer a higher dose of the drug. The subsequent increase in drug plasma concentrations leads to toxicity, making this approach impractical (e.g., fluoroquinolones) [77]. Another possibility is intrathecal antibiotic administration, an invasive technique with the drawback of high patient variability [77]. However, liposomes have been investigated to overcome this issue, as alterations to the liposomal surface can improve the BBB penetration. Altering the surface properties of liposomes can increase drug delivery across the BBB, enhancing drug deposition into the central nervous system. For example, receptor-mediated transcytosis with monoclonal antibodies to transferrin receptors and monoclonal antibodies to insulin receptors has been used to demonstrate internalization into brain endothelial cells in animal models [78,79].

Niosomes

Non-ionic surfactant vesicles are being utilized for various applications, ranging from oral, transdermal, and topical, as well as for creating dosage forms that can penetrate the brain barrier. Structurally, they resemble liposomes, but instead of phospholipids, they are vesicular structures prepared from non-ionic surfactants (sorbitan esters (span series) and polysorbates (tween series), cholesterol, and fatty alcohols). Niosomes have been used in cosmetic applications since the 1970s, and their non-toxic and non-immunogenic nature makes them good candidates for targeted drug delivery. They have numerous advantages when compared to liposomes and exhibit superior physical and chemical stability, while also being more cost-effective [80]. These surfactants are regarded as safe, as the potential for toxicity is lower compared to anionic and cationic surfactants. The linkage between the two parts may be an amide, ether, or ester, with alkyl ethers being the most commonly used. The hydrophilic head may be derived from glycerol or large sugar molecules, or ethylene oxide subunits. Monoalkyl (from C12 to C18) or dialkyl chains may be used for the hydrophobic tail. They are osmotically active, and the vesicles show greater stability than liposomes. Incorporation of cholesterol adds rigidity to the bilayer membrane, improving its stability. Unlike liposomes, which are prepared with phospholipids that require special handling during their preparation, such as maintaining a nitrogen atmosphere to prevent oxidation of phospholipids, the components of niosomes are more stable and are not subjected to oxidative degradation. Niosomes have numerous advantages over liposomes, such as simple manufacturing methods and low production costs. Another difference between liposomes and niosomes is that the use of phospholipids makes liposomes more readily phagocytosed than niosomes prepared with non-ionic surfactants, resulting in a longer residence time. The vesicle surface can be manipulated to allow for targeted delivery, which enhances drug efficacy as the clearance is reduced [81]. The surfactants are classified as biodegradable, biocompatible, and nonimmunogenic, which makes them suitable for use in humans.

Antibiotics Encapsulated in Niosomes

Therapeutic failure is common for some conventional drug treatments due to limited permeation of the antimicrobial agent, as well as the larger volume of distribution, which results in accumulation of the drug at sites where its action is not required. Antibiotic encapsulation in niosomes has the potential for reducing the extent of drug distribution while delivering the drug to the site of infection. Once administered, niosomes can be engulfed by phagocytes, which provides a highly efficient method of drug delivery to the infection site, thereby reducing the volume of distribution and chances of the development of toxicity [82].

Niosomal Antibiotics for Ophthalmic Drug Delivery

Drug therapy in ocular delivery is challenging because of the short contact time of the drug with the eye surfaces, drug loss via nasolacrimal drainage, and corneal impermeability. Another factor that influences drug efficacy is compliance, which decreases with frequent instillation. To circumvent these challenges, newer ocular delivery systems are being investigated [83,84], such as niosomal formulations of gentamicin [85]. These systems increase the drug-corneal surface interaction time, prevent degradation of the drug, and provide sustained action at the corneal epithelial surface. Commonly used antibiotics that have been encapsulated in niosomes for in vitro/in vivo evaluation are shown in Table 2.

**Table 2:** Commonly used antibiotics that have been encapsulated in niosomes for in vitro/in vivo evaluation. MIC = minimum inhibitory concentration; MRSA = methicillin-resistant Staphylococcus aureus

Antibiotic	Model	Major findings	Reference
Streptomycin	To investigate the antibacterial effects of streptomycin-loaded niosomes and their ability to reduce biofilm formation	Superior activity against *Staphylococcus aureus*, *Escherichia coli*, and *Pseudomonas aeruginosa* with 4–8-fold MIC reduction. No toxicity observed in human fibroblasts at 1,500 µg/mL	[86]
Tobramycin	To determine the effect of tobramycin encapsulated in niosomes against clinical strains of *Pseudomonas aeruginosa* resistant to multiple antibiotics	Enhanced antibacterial activity via downregulation of MexAB-OprM efflux and biofilm-related genes	[87]
Amikacin	To evaluate niosomal amikacin in reducing biofilm formation in resistant *Klebsiella* strains via the *mrkD* gene expression	Niosomal amikacin showed reduced cytotoxicity and significantly decreased *mrkD* mRNA expression compared to free amikacin	[88]
Gentamycin	To investigate niosome-entrapped gentamycin sulfate as a potential drug delivery system	Sustained release observed; no ocular irritation in albino rabbits.	[85]
Vancomycin	To assess the physiological properties of vancomycin-loaded niosomes and their antimicrobial potential	pH-sensitive gel with >46% encapsulation efficiency; enhanced antibacterial efficacy in vitro and in vivo with >24-hour release	[89]
Lomefloxacin	To evaluate the drug release and antimicrobial properties of lomefloxacin niosomes	Sustained release for up to 8 hours; 35-fold higher antibacterial activity compared to free drug in vivo	[90]
Azithromycin	To investigate niosomal azithromycin prepared using azithromycin–β-cyclodextrin complex	Encapsulation efficiency >65%; enhanced corneal penetration and extended 12-hour drug release. Effective for bacterial conjunctivitis	[91]
Natamycin	To examine niosomal natamycin properties and delivery profile as an in situ ophthalmic gel system	Encapsulation efficiency >65%; extended release up to 24 hours, improved corneal permeation, and no irritation or redness	[92]
Doxycycline	To investigate the efficacy of doxycycline encapsulated into niosomes using cholesterol with Spans and Tweens in varying molar ratios	Encapsulation efficiency >50%; extended release for >20 hours with minimal toxicity. Suitable for ocular surface disease treatment	[93]
Imipenem	To examine niosomal imipenem on MRSE biofilm gene expression	Improved antimicrobial activity and reduced biofilm formation tendency	[94]
Carbapenem	To evaluate surfactant vesicles of ertapenem, meropenem, and tigecycline against Gram-negative isolates	Niosomal meropenem showed up to 5-fold, ertapenem 1–9-fold, and tigecycline 1–7-fold MIC reductions	[95]
Ciprofloxacin	To investigate ciprofloxacin niosomes against resistant *Staphylococcus aureus* strains	Enhanced antibacterial activity with reduced MIC and *icaB* gene inhibition	[96]
Levofloxacin	To evaluate niosomal levofloxacin in a rat model of Pseudomonas aeruginosa peritonitis and assess the toxicity of Span 60 vesicles	Reduced bacterial counts in major organs and lower lymphocyte/neutrophil counts; no signs of toxicity observed	[97][98]
Cefazolin	To examine cefazolin-loaded niosomes against MRSA from skin/soft tissue infections	Successfully removed biofilms at 128–256 µg/mL; faster recovery observed in BALB/c mice compared to free drug	[99]

These formulations are cost-effective, easy to fabricate, provide superior physical stability, and improve bioavailability with a reduced likelihood of blurring of vision. The formulations fulfil the requirements of an ophthalmic drug delivery system with localized and long duration of action. Niosomes also allow for enhanced drug levels in the anterior and posterior eye segments through improved corneal penetration. The advantages of niosomes can be further improved by coating the surface with mucoadhesive polymers such as chitosan, Carbopol, or hyaluronic acid. Niosomes used for in situ gels showed no signs of irritation, inflammation, or redness.

Clinical Trials

Several trials conducted with nanoparticles have yielded promising results. One such scenario is the critical need for more effective therapeutic strategies for individuals with non-tuberculous mycobacterial pulmonary disease caused by *M. avium* complex, which is unresponsive to standard treatment. Incorporating amikacin liposome inhalation suspension into guideline-based therapy for these refractory cases resulted in a markedly higher rate of culture conversion at six months compared to guideline-based therapy alone, without an increase in serious adverse events [100].

Pegylated liposomal dexamethasone phosphate (Dex-PL) demonstrated favorable safety and tolerability in a phase I trial involving multiple myeloma patients. This trial suggested increased circulating time, enhanced drug delivery, and reduced adverse effect profile of liposomal formulations. Compared to conventional dexamethasone, Dex-PL maintained high circulating drug levels for over a week, likely reducing systemic exposure and adverse effects. These findings suggest Dex-PL may offer a safer, more effective corticosteroid option pending confirmation in larger studies [101].

Pegylated liposomal doxorubicin (PEG-LD) combined with cisplatin showed acceptable safety and promising activity in metastatic or recurrent osteosarcoma. In a phase I trial, the maximum tolerated dose was 50 mg/m², with manageable toxicities, primarily hematologic. The regimen achieved a 13.3% response rate and 66.7% disease control, supporting further evaluation in phase II studies [102].

In the ROLANDO phase II trial, PEG-LD combined with olaparib showed clinical activity in platinum-resistant ovarian cancer. The median overall survival reached 21.3 months in favorable prognostic clusters. High neutrophil/lymphocyte ratio predicted poorer outcomes, while a multifactorial baseline signature provided better prognostic accuracy. These findings support personalized treatment strategies and warrant validation in larger cohorts [103].

A phase 1 trial evaluated the safety and feasibility of enhancing doxorubicin delivery to liver tumors using lyso-thermosensitive liposomal doxorubicin, combined with non-invasive focused ultrasound hyperthermia. Ten patients received treatment, achieving a mean 3.7-fold increase in intratumoral drug concentration. The approach was well tolerated, with expected hematologic toxicity, and demonstrated potential for targeted chemotherapy in unresectable liver tumors [104].

Nanoliposomal irinotecan was evaluated in a global phase 3 trial for metastatic pancreatic ductal adenocarcinoma after gemcitabine therapy. Among 417 patients, the combination of nanoliposomal irinotecan with fluorouracil and folinic acid improved median overall survival to 6.1 months versus 4.2 months with control, while monotherapy showed no benefit. Toxicities were manageable, supporting this regimen as a new treatment option [105].

Non-tuberculous mycobacteria cause persistent lung infections, often challenging to diagnose and treat. *M. avium* complex and *M. abscessus* dominate among pathogenic species. Recently, inhaled amikacin (Arikayce) demonstrated efficacy in refractory non-tuberculous mycobacteria cases, achieving sputum culture conversion in 29% versus 8.9% with standard therapy. Further trials are required to confirm safety, optimal candidates, and long-term benefits [106].

Safety Concerns and Evaluation of Toxicity of Niosome Components

The European Union (EU) re-evaluated the use of Sorbitan esters as food additives in 2017. In 1974, the Scientific Committee on Food reported that the daily consumption of sorbitan esters was safe at 25 mg/kg. When sorbitan monostearate is ingested, it is hydrolyzed and eventually eliminated in urine, faeces, or expelled as CO₂. The Panel considered that there was no concern for genotoxicity associated with the use of these esters. The Panel suggested an acceptable daily intake of 10 mg/kg for sorbitan esters, which is equivalent to 26 mg sorbitan monostearate/kg per day [107]. The in vivo toxicity of sorbitan esters in *P. aeruginosa*-infected Sprague Dawley rats was investigated, and necropsy performed 14 days after treatment showed no discernible effect on body organs [98].

Biomimetic Nanovesicular Particles

Biomimetic nanovesicular particles (BNPs) exploit the properties of biological components of membranes of red blood cells, cancer cells, bacterial cells, and exosomes. They are biocompatible, highly specific, have long circulation time, and low immunogenicity. A recently employed method used natural membranes, such as Gram-negative organisms’ outer membrane vesicles (OMVs), as carriers of antibiotics to infected sites. Unlike nanoparticles, these vesicles have a hollow hydrophilic interior similar to liposomes and can be used to encapsulate drugs. The OMVs contain proteins in addition to lipids. These natural vesicles are biocompatible and non-immunogenic. No chemical steps are required for their production; only the cell culture and growth conditions need to be optimized. *E. coli*-derived OMV loaded with rifampicin elevated the intracellular concentration of the drug, but was not effective against *S. aureus*, as it cannot permeate through the double membrane structure. OMVs derived from *A. baumannii* that contained fluoroquinolones were able to enter Gram-negative bacterial cells of multidrug-resistant organisms such as *P. aeruginosa* and *K. pneumoniae* strains, and induced bacterial cell death in vitro and in vivo [108]. Biological vectors show promise as an option for imaging and therapeutics when targeting specificity is required.

## Conclusions

Since the search for new antibiotics has slowed down, alternative solutions are required to fight against drug-resistant bacteria. Improving the current antibiotic delivery systems may be an option, as recent developments in nanomedicine are leading the way to develop new drug carriers for antibiotics. A promising method is the encapsulation of the antibiotic in lipid-based formulations such as liposomes, niosomes, and other similar nanovesicular systems. Encapsulation of the antibiotic in nanovesicular systems is relatively easy to accomplish and results in increased antibiotic concentrations at the infection site and improved permeability, biodistribution, and pharmacokinetics. The antibiotic is also protected from degradation en route to the infection site, thus ensuring stability and minimizing toxicity to healthy tissues. Several antibiotics enclosed in liposomes have already been introduced in clinical practice. However, liposomes do have stability problems such as aggregation and fusion of vesicles, leakage of the encapsulated drug, oxidation and hydrolysis of lipid components, complex manufacturing processes, limited encapsulation efficiency, rapid clearance, immunogenicity, and regulatory hurdles. In addition, maintaining quality control from batch-to-batch production, surface charge, stability, particle size, and size distribution of the nanoparticles is a challenge. Despite promising advances in liposome technology, these challenges must be addressed to fully realize the potential of antibiotic-loaded nanovesicular systems in clinical practice. Ongoing research and technological innovations continue to offer hope of finding solutions to fight against fatal multidrug-resistant bacterial infections.

## References

[1] Dadgostar P (2019). Antimicrobial resistance: implications and costs. Infect Drug Resist.

[2] (2025). Global Antimicrobial Resistance and Use Surveillance System (GLASS) report. https://www.who.int/publications/i/item/9789241564748.

[3] (2025). Global Antimicrobial Resistance and Use Surveillance System (GLASS) report 2022. https://iris.who.int/bitstream/handle/10665/364996/9789240062702-eng.pdf.

[4] Munita JM, Arias CA (2016). Mechanisms of antibiotic resistance. Microbiol Spectr.

[5] Tang K, Zhao H (2023). Quinolone antibiotics: resistance and therapy. Infect Drug Resist.

[6] Correia S, Poeta P, Hébraud M, Capelo JL, Igrejas G (2017). Mechanisms of quinolone action and resistance: where do we stand?. J Med Microbiol.

[7] McMurry L, Petrucci RE Jr, Levy SB (1980). Active efflux of tetracycline encoded by four genetically different tetracycline resistance determinants in Escherichia coli. Proc Natl Acad Sci U S A.

[8] Poole K (2005). Efflux-mediated antimicrobial resistance. J Antimicrob Chemother.

[9] Eaves DJ, Ricci V, Piddock LJ (2004). Expression of acrB, acrF, acrD, marA, and soxS in Salmonella enterica serovar Typhimurium: role in multiple antibiotic resistance. Antimicrob Agents Chemother.

[10] Nishino K, Yamaguchi A (2001). Analysis of a complete library of putative drug transporter genes in Escherichia coli. J Bacteriol.

[11] De Angelis G, Del Giacomo P, Posteraro B, Sanguinetti M, Tumbarello M (2020). Molecular mechanisms, epidemiology, and clinical importance of β-lactam resistance in Enterobacteriaceae. Int J Mol Sci.

[12] Yassien M, Khardori N, Ahmedy A, Toama M (1995). Modulation of biofilms of Pseudomonas aeruginosa by quinolones. Antimicrob Agents Chemother.

[13] Uruén C, Chopo-Escuin G, Tommassen J, Mainar-Jaime RC, Arenas J (2020). Biofilms as promoters of bacterial antibiotic resistance and tolerance. Antibiotics (Basel).

[14] Olsen I (2015). Biofilm-specific antibiotic tolerance and resistance. Eur J Clin Microbiol Infect Dis.

[15] Morck DW, Lam K, McKay SG (1994). Comparative evaluation of fleroxacin, ampicillin, trimethoprimsulfamethoxazole, and gentamicin as treatments of catheter-associated urinary tract infection in a rabbit model. Int J Antimicrob Agents.

[16] Ceri H, Olson ME, Stremick C, Read RR, Morck D, Buret A (1999). The Calgary Biofilm Device: new technology for rapid determination of antibiotic susceptibilities of bacterial biofilms. J Clin Microbiol.

[17] Bhumireddy SK, Gudla SS, Vadaga AK, Nandula MS (2025). Vancomycin-induced DRESS syndrome: a systematic review of case reports. Hosp Pharm.

[18] Viegas C, Patrício AB, Prata JM, Nadhman A, Chintamaneni PK, Fonte P (2023). Solid lipid nanoparticles vs. nanostructured lipid carriers: a comparative review. Pharmaceutics.

[19] Unnikrishnan G, Joy A, Megha M, Kolanthai E, Senthilkumar M (2023). Exploration of inorganic nanoparticles for revolutionary drug delivery applications: a critical review. Discov Nano.

[20] Gimondi S, Ferreira H, Reis RL, Neves NM (2023). Microfluidic devices: a tool for nanoparticle synthesis and performance evaluation. ACS Nano.

[21] Murugaiyan J, Kumar PA, Rao GS (2022). Progress in alternative strategies to combat antimicrobial resistance: focus on antibiotics. Antibiotics (Basel).

[22] Hetta HF, Ramadan YN, Al-Harbi AI (2023). Nanotechnology as a promising approach to combat multidrug resistant bacteria: a comprehensive review and future perspectives. Biomedicines.

[23] Allen TM (1997). Liposomes. Opportunities in drug delivery. Drugs.

[24] Malhotra M, Jain NK (1994). Niosomes as drug carriers. Indian Drugs.

[25] Rinaldi F, Hanieh PN, Sennato S (2021). Rifampicin-liposomes for Mycobacterium abscessus infection treatment: intracellular uptake and antibacterial activity evaluation. Pharmaceutics.

[26] Gaspar MM, Cruz A, Penha AF (2008). Rifabutin encapsulated in liposomes exhibits increased therapeutic activity in a model of disseminated tuberculosis. Int J Antimicrob Agents.

[27] Pinho JO, Ferreira M, Coelho M, Pinto SN, Aguiar SI, Gaspar MM (2024). Liposomal rifabutin-A promising antibiotic repurposing strategy against methicillin-resistant Staphylococcus aureus infections. Pharmaceuticals (Basel).

[28] Ferreira M, Ogren M, Dias JN (2021). Liposomes as antibiotic delivery systems: a promising nanotechnological strategy against antimicrobial resistance. Molecules.

[29] Fu YY, Zhang L, Yang Y, Liu CW, He YN, Li P, Yu X (2019). Synergistic antibacterial effect of ultrasound microbubbles combined with chitosan-modified polymyxin B-loaded liposomes on biofilm-producing Acinetobacter baumannii. Int J Nanomedicine.

[30] Nicolosi D, Cupri S, Genovese C, Tempera G, Mattina R, Pignatello R (2015). Nanotechnology approaches for antibacterial drug delivery: preparation and microbiological evaluation of fusogenic liposomes carrying fusidic acid. Int J Antimicrob Agents.

[31] Nicolosi D, Scalia M, Nicolosi VM, Pignatello R (2010). Encapsulation in fusogenic liposomes broadens the spectrum of action of vancomycin against Gram-negative bacteria. Int J Antimicrob Agents.

[32] Mugabe C, Halwani M, Azghani AO, Lafrenie RM, Omri A (2006). Mechanism of enhanced activity of liposome-entrapped aminoglycosides against resistant strains of Pseudomonas aeruginosa. Antimicrob Agents Chemother.

[33] Rukholm G, Mugabe C, Azghani AO, Omri A (2006). Antibacterial activity of liposomal gentamicin against Pseudomonas aeruginosa: a time-kill study. Int J Antimicrob Agents.

[34] Ribeiro LN, de Paula E, Rossi DA (2020). Hybrid pectin-liposome formulation against multi-resistant bacterial strains. Pharmaceutics.

[35] Furneri PM, Fresta M, Puglisi G, Tempera G (2000). Ofloxacin-loaded liposomes: in vitro activity and drug accumulation in bacteria. Antimicrob Agents Chemother.

[36] Moyá ML, López-López M, Lebrón JA (2019). Preparation and characterization of new liposomes. Bactericidal activity of cefepime encapsulated into cationic liposomes. Pharmaceutics.

[37] Vanić Ž, Rukavina Z, Manner S (2019). Azithromycin-liposomes as a novel approach for localized therapy of cervicovaginal bacterial infections. Int J Nanomedicine.

[38] Webb MS, Boman NL, Wiseman DJ (1998). Antibacterial efficacy against an in vivo Salmonella typhimurium infection model and pharmacokinetics of a liposomal ciprofloxacin formulation. Antimicrob Agents Chemother.

[39] Nacucchio MC, Bellora MJ, Sordelli DO, D'Aquino M (1985). Enhanced liposome-mediated activity of piperacillin against staphylococci. Antimicrob Agents Chemother.

[40] Sande L, Sanchez M, Montes J, Wolf AJ, Morgan MA, Omri A, Liu GY (2012). Liposomal encapsulation of vancomycin improves killing of methicillin-resistant Staphylococcus aureus in a murine infection model. J Antimicrob Chemother.

[41] Sang N, Jiang L, Wang Z, Zhu Y, Lin G, Li R, Zhang J (2022). Bacteria-targeting liposomes for enhanced delivery of cinnamaldehyde and infection management. Int J Pharm.

[42] Jubrail J, Morris P, Bewley MA (2016). Inability to sustain intraphagolysosomal killing of Staphylococcus aureus predisposes to bacterial persistence in macrophages. Cell Microbiol.

[43] Carryn S, Chanteux H, Seral C, Mingeot-Leclercq MP, Van Bambeke F, Tulkens PM (2003). Intracellular pharmacodynamics of antibiotics. Infect Dis Clin North Am.

[44] Tulkens PM (1991). Intracellular distribution and activity of antibiotics. Eur J Clin Microbiol Infect Dis.

[45] Abed N, Couvreur P (2014). Nanocarriers for antibiotics: a promising solution to treat intracellular bacterial infections. Int J Antimicrob Agents.

[46] Tulkens P, Trouet A (1978). The uptake and intracellular accumulation of aminoglycoside antibiotics in lysosomes of cultured rat fibroblasts. Biochem Pharmacol.

[47] al-Nawas B, Shah PM (1998). Intracellular activity of vancomycin and Ly333328, a new semisynthetic glycopeptide, against methicillin-resistant Staphylococcus aureus. Infection.

[48] Bastos MD, Coutinho BG, Coelho ML (2010). Lysostaphin: a staphylococcal bacteriolysin with potential clinical applications. Pharmaceuticals (Basel).

[49] Wu JA, Kusuma C, Mond JJ, Kokai-Kun JF (2003). Lysostaphin disrupts Staphylococcus aureus and Staphylococcus epidermidis biofilms on artificial surfaces. Antimicrob Agents Chemother.

[50] Gründling A, Schneewind O (2006). Cross-linked peptidoglycan mediates lysostaphin binding to the cell wall envelope of Staphylococcus aureus. J Bacteriol.

[51] Hajiahmadi F, Alikhani MY, Shariatifar H, Arabestani MR, Ahmadvand D (2019). The bactericidal effect of lysostaphin coupled with liposomal vancomycin as a dual combating system applied directly on methicillin-resistant Staphylococcus aureus infected skin wounds in mice. Int J Nanomedicine.

[52] Zarogoulidis P, Kioumis I, Porpodis K (2013). Clinical experimentation with aerosol antibiotics: current and future methods of administration. Drug Des Devel Ther.

[53] Khatib I, Khanal D, Ruan J, Cipolla D, Dayton F, Blanchard JD, Chan HK (2019). Ciprofloxacin nanocrystals liposomal powders for controlled drug release via inhalation. Int J Pharm.

[54] Bassetti M, Vena A, Russo A, Peghin M (2020). Inhaled liposomal antimicrobial delivery in lung infections. Drugs.

[55] Liu C, Shi J, Dai Q, Yin X, Zhang X, Zheng A (2015). In-vitro and in-vivo evaluation of ciprofloxacin liposomes for pulmonary administration. Drug Dev Ind Pharm.

[56] Serisier DJ (2012). Inhaled antibiotics for lower respiratory tract infections: focus on ciprofloxacin. Drugs Today (Barc).

[57] Wang S, Yu S, Lin Y (2018). Co-delivery of ciprofloxacin and colistin in liposomal formulations with enhanced in vitro antimicrobial activities against multidrug resistant Pseudomonas aeruginosa. Pharm Res.

[58] Islam N, Reid D (2024). Inhaled antibiotics: a promising drug delivery strategies for efficient treatment of lower respiratory tract infections (LRTIs) associated with antibiotic resistant biofilm-dwelling and intracellular bacterial pathogens. Respir Med.

[59] Lipsky BA, Hoey C (2009). Topical antimicrobial therapy for treating chronic wounds. Clin Infect Dis.

[60] Wang W, Lu KJ, Yu CH, Huang QL, Du YZ (2019). Nano-drug delivery systems in wound treatment and skin regeneration. J Nanobiotechnology.

[61] Price CI, Horton JW, Baxter CR (1990). Topical liposomal delivery of antibiotics in soft tissue infection. J Surg Res.

[62] Augustin M, Goepel L, Jacobi A, Bosse B, Mueller S, Hopp M (2017). Efficacy and tolerability of liposomal polyvinylpyrrolidone-iodine hydrogel for the localized treatment of chronic infective, inflammatory, dermatoses: an uncontrolled pilot study. Clin Cosmet Investig Dermatol.

[63] Ferreira M, Aguiar S, Bettencourt A, Gaspar MM (2021). Lipid-based nanosystems for targeting bone implant-associated infections: current approaches and future endeavors. Drug Deliv Transl Res.

[64] Drulis-Kawa Z, Dorotkiewicz-Jach A, Gubernator J, Gula G, Bocer T, Doroszkiewicz W (2009). The interaction between Pseudomonas aeruginosa cells and cationic PC:Chol:DOTAP liposomal vesicles versus outer-membrane structure and envelope properties of bacterial cell. Int J Pharm.

[65] Liu Y, Sun D, Fan Q (2020). The enhanced permeability and retention effect based nanomedicine at the site of injury. Nano Res.

[66] Nisini R, Poerio N, Mariotti S, De Santis F, Fraziano M (2018). The multirole of liposomes in therapy and prevention of infectious diseases. Front Immunol.

[67] Kneidl B, Peller M, Winter G, Lindner LH, Hossann M (2014). Thermosensitive liposomal drug delivery systems: state of the art review. Int J Nanomedicine.

[68] Alhariri M, Azghani A, Omri A (2013). Liposomal antibiotics for the treatment of infectious diseases. Expert Opin Drug Deliv.

[69] Yang X, Xie B, Peng H (2021). Eradicating intracellular MRSA via targeted delivery of lysostaphin and vancomycin with mannose-modified exosomes. J Control Release.

[70] Santos-Ferreira I, Bettencourt A, Almeida AJ (2015). Nanoparticulate platforms for targeting bone infections: meeting a major therapeutic challenge. Nanomedicine (Lond).

[71] Soares D, Leite P, Barreira P, Aido R, Sousa R (2015). Antibiotic-loaded bone cement in total joint arthroplasty. Acta Orthop Belg.

[72] Jiranek WA, Hanssen AD, Greenwald AS (2006). Antibiotic-loaded bone cement for infection prophylaxis in total joint replacement. J Bone Joint Surg Am.

[73] Bistolfi A, Massazza G, Verné E (2011). Antibiotic-loaded cement in orthopedic surgery: a review. ISRN Orthop.

[74] Ferreira M, Rzhepishevska O, Grenho L (2017). Levofloxacin-loaded bone cement delivery system: highly effective against intracellular bacteria and Staphylococcus aureus biofilms. Int J Pharm.

[75] Snoddy B, Jayasuriya AC (2016). The use of nanomaterials to treat bone infections. Mater Sci Eng C Mater Biol Appl.

[76] Hui T, Yongqing X, Tiane Z (2009). Treatment of osteomyelitis by liposomal gentamicin-impregnated calcium sulfate. Arch Orthop Trauma Surg.

[77] Nau R, Sörgel F, Eiffert H (2010). Penetration of drugs through the blood-cerebrospinal fluid/blood-brain barrier for treatment of central nervous system infections. Clin Microbiol Rev.

[78] Loureiro JA, Gomes B, Fricker G (2015). Dual ligand immunoliposomes for drug delivery to the brain. Colloids Surf B Biointerfaces.

[79] Li X, Tsibouklis J, Weng T (2017). Nano carriers for drug transport across the blood-brain barrier. J Drug Target.

[80] Bartelds R, Nematollahi MH, Pols T, Stuart MC, Pardakhty A, Asadikaram G, Poolman B (2018). Niosomes, an alternative for liposomal delivery. PLoS One.

[81] Adepu S, Ramakrishna S (2021). Controlled drug delivery systems: current status and future directions. Molecules.

[82] Su S, M Kang P (2020). Recent advances in nanocarrier-assisted therapeutics delivery systems. Pharmaceutics.

[83] Durak S, Esmaeili Rad M, Alp Yetisgin A, Eda Sutova H, Kutlu O, Cetinel S, Zarrabi A (2020). Niosomal drug delivery systems for ocular disease-recent advances and future prospects. Nanomaterials (Basel).

[84] Sahoo RK, Biswas N, Guha A, Sahoo N, Kuotsu K (2014). Nonionic surfactant vesicles in ocular delivery: innovative approaches and perspectives. Biomed Res Int.

[85] Abdelbary G, El-Gendy N (2008). Niosome-encapsulated gentamicin for ophthalmic controlled delivery. AAPS PharmSciTech.

[86] Mansouri M, Khayam N, Jamshidifar E (2021). Streptomycin sulfate-loaded niosomes enables increased antimicrobial and anti-biofilm activities. Front Bioeng Biotechnol.

[87] Hedayati Ch M, Abolhassani Targhi A, Shamsi F (2021). Niosome-encapsulated tobramycin reduced antibiotic resistance and enhanced antibacterial activity against multidrug-resistant clinical strains of Pseudomonas aeruginosa. J Biomed Mater Res A.

[88] Rahmati M, Babapoor E, Dezfulian M (2022). Amikacin-loaded niosome nanoparticles improve amikacin activity against antibiotic-resistant Klebsiella pneumoniae strains. World J Microbiol Biotechnol.

[89] Allam A, El-Mokhtar MA, Elsabahy M (2019). Vancomycin-loaded niosomes integrated within pH-sensitive in-situ forming gel for treatment of ocular infections while minimizing drug irritation. J Pharm Pharmacol.

[90] Abdelbary A, Salem HF, Khallaf RA, Ali AM (2017). Mucoadhesive niosomal in situ gel for ocular tissue targeting: in vitro and in vivo evaluation of lomefloxacin hydrochloride. Pharm Dev Technol.

[91] Akhtar N, Kumar Singh R, Pathak K (2017). Exploring the potential of complex-vesicle based niosomal ocular system loaded with azithromycin: development of in situ gel and ex vivo characterization. Pharm Biomed Res.

[92] Paradkar MU, Parmar M (2017). Formulation development and evaluation of Natamycin niosomal in-situ gel for ophthalmic drug delivery. J Drug Deliv Sci Technol.

[93] Gugleva V, Titeva S, Rangelov S, Momekova D (2019). Design and in vitro evaluation of doxycycline hyclate niosomes as a potential ocular delivery system. Int J Pharm.

[94] Piri-Gharaghie T, Jegargoshe-Shirin N, Saremi-Nouri S (2022). Effects of imipenem-containing niosome nanoparticles against high prevalence methicillin-resistant Staphylococcus epidermidis biofilm formed. Sci Rep.

[95] Abo Kamer AM, Amer NM, Abdelmegeed AA, El Maghraby GM, Gamaleldin NM (2023). Surfactant nanovesicles for augmented antibacterial activity against carbapenemase resistant enterobacteriaceae and extended spectrum beta-lactamases producing bacteria: in vitro and in vivo evaluation. BMC Microbiol.

[96] Mirzaie A, Peirovi N, Akbarzadeh I (2020). Preparation and optimization of ciprofloxacin encapsulated niosomes: a new approach for enhanced antibacterial activity, biofilm inhibition and reduced antibiotic resistance in ciprofloxacin-resistant methicillin-resistance Staphylococcus aureus. Bioorg Chem.

[97] Jankie S, Johnson J, Adebayo AS, Pillai GK, Pinto Pereira LM (2020). Efficacy of levofloxacin loaded nonionic surfactant vesicles (niosomes) in a model of Pseudomonas aeruginosa infected Sprague Dawley rats. Adv Pharmacol Pharm Sci.

[98] Jankie S, Johnson J, Adebayo AS, Pillai GK, Pinto Pereira LM (2016). Acute and subacute toxicity of sorbitan monostearate (Span 60) non-ionic surfactant vesicles (niosomes) in Sprague Dawley rats. J Pharm Res Int.

[99] Zafari M, Adibi M, Chiani M (2021). Effects of cefazolin-containing niosome nanoparticles against methicillin-resistant Staphylococcus aureus biofilm formed on chronic wounds. Biomed Mater.

[100] Griffith DE, Eagle G, Thomson R (2018). Amikacin liposome inhalation suspension for treatment-refractory lung disease caused by Mycobacterium avium complex (CONVERT). A prospective, open-label, randomized study. Am J Respir Crit Care Med.

[101] Metselaar J, Lammers T, Boquoi A (2023). A phase I first-in-man study to investigate the pharmacokinetics and safety of liposomal dexamethasone in patients with progressive multiple myeloma. Drug Deliv Transl Res.

[102] Wen XZ, Pan QZ, Xu BS (2022). Phase I study of pegylated liposomal doxorubicin and cisplatin in patients with advanced osteosarcoma. Cancer Chemother Pharmacol.

[103] Perez-Fidalgo JA, Guerra E, García Y (2023). Clinical and molecular signature of survival and resistance to olaparib plus pegylated liposomal doxorubicin in platinum-resistant ovarian cancer: a stratified analysis from the phase II clinical trial ROLANDO, GEICO-1601. Int J Gynecol Cancer.

[104] Lyon PC, Gray MD, Mannaris C (2018). Safety and feasibility of ultrasound-triggered targeted drug delivery of doxorubicin from thermosensitive liposomes in liver tumours (TARDOX): a single-centre, open-label, phase 1 trial. Lancet Oncol.

[105] Wang-Gillam A, Li CP, Bodoky G (2016). Nanoliposomal irinotecan with fluorouracil and folinic acid in metastatic pancreatic cancer after previous gemcitabine-based therapy (NAPOLI-1): a global, randomised, open-label, phase 3 trial. Lancet.

[106] Khan O, Chaudary N (2020). The use of amikacin liposome inhalation suspension (Arikayce) in the treatment of refractory nontuberculous mycobacterial lung disease in adults. Drug Des Devel Ther.

[107] Mortensen A, Aguilar F, Crebelli R (2017). Re-evaluation of sorbitan monostearate (E 491), sorbitan tristearate (E 492), sorbitan monolaurate (E 493), sorbitan monooleate (E 494) and sorbitan monopalmitate (E 495) when used as food additives. EFSA J.

[108] Huang W, Zhang Q, Li W (2020). Development of novel nanoantibiotics using an outer membrane vesicle-based drug efflux mechanism. J Control Release.

